# Risk factors associated with acute hepatopancreatic necrosis disease at shrimp farm level in Bac Lieu Province, Vietnam

**DOI:** 10.14202/vetworld.2021.1050-1058

**Published:** 2021-04-30

**Authors:** Hien The Nguyen, Toan Nguyen Van, Tien Tien Ngoc, Visanu Boonyawiwat, Theera Rukkwamsuk, Adisorn Yawongsa

**Affiliations:** 1 Department of Animal Health of Vietnam, No. 15 lane 78, Giai Phong Street, Phuong Mai Ward, Dong Da District, Hanoi, Vietnam; 2 Faculty of Veterinary Medicine, Kasetsart University, No. 50, Phahonyothin Road, Ladyao, Chatuchak, Bangkok, Thailand; 3 Sub-Department of Livestock Production and Animal Health of Bac Lieu Province, No. 217, 23/8 Road, 8^th^ Ward, Bac Lieu City, Bac Lieu province, Vietnam; 4 Regional Animal Health Office number VII, No. 88 Cach Mang Thang 8 Street, Cai Khe Ward, Binh Thuy District, Can Tho City, Vietnam

**Keywords:** conditional logistic regression, early mortality syndrome, fish-eating bird, matched case-control study, odd ratio

## Abstract

**Background and Aim:**

Acute hepatopancreatic necrosis disease (AHPND) is a severe disease in shrimp farms and adversely affected the shrimp industry of Vietnam. So far, the study on risk factors associated with AHPND outbreaks is limited. The objective of this study was to determine the potential risk factors of AHPND at the shrimp farm level in Bac Lieu Province, Vietnam.

**Materials and Methods:**

Real-time-Polymerase chain reaction was used to analyze data collected from an active surveillance program of shrimp farms in 2017 in the Vinh Tien and Vinh Lac villages, Vinh Thinh commune, Hoa Binh district in Bac Lieu Province, Vietnam. The matched case-control study selected 20 cases and 20 control farms from 134 shrimp farms. In 2018, face-to-face interviews using structured questionnaires were conducted with the farmers of these selected farms.

**Results:**

Of the 59 studied variables, seven had p≤0.2 based on bivariate analyses. The results of multivariable analysis showed that the presence of fish-eating birds on shrimp farms was a significant association with AHPND (odds ratio=8, p=0.049).

**Conclusion:**

To reduce the effect of AHPND, farmers should apply effective methods to manage wild animals such as using a grid or net to cover the pond, combined with improved biosecurity.

## Introduction

Acute hepatopancreatic necrosis disease (AHPND) is a severe disease in shrimp farms and specifically has caused great economic losses and adversely affected the shrimp industry of Vietnam. AHPND was firstly recognized in Vietnam in Soc Trang Province on the Mekong River Delta (MRD) at the end of 2010. In 2011, AHPND continued to spread to other provinces such as Tien Giang, Ben Tre, Tra Vinh, Soc Trang, Bac Lieu, Kien Giang, and Ca Mau Provinces [1-3] covering a total shrimp pond area of approximately 98,000 ha. By 2017, AHPND had spread to 294 communes in 86 districts of 25 provinces throughout the country [4]. Thus, prevention and control of AHPND have become priorities. AHPND is called early mortality syndrome in brackish shrimps. The first case was reported in 2009 in China [5] and this disease spread quickly to Vietnam in 2010, Malaysia in 2011, Thailand in 2012, Mexico in 2013, and the Philippines in 2014 [2,6-10]. AHPND was later detected in Central America in Ecuador and it is expected to continue spreading [11].

The identification of risk factors is necessary to prevent and control AHPND effectively. However, the lack of field studies on risk factors has been a limitation. A study was conducted in four districts on the MRD of Vietnam under the TCP/VIE/3304 project of Food and Agriculture Organization (FAO) during 2012-2013. The results showed that the main risk factors at the farm level were: Large farm size, using the sun-dried sediment method, farm location nearby other farms, and using a water source already affected by AHPND [12]. EHP infection was also identified as risk factor for AHPND [13]. However, there has been no research published to confirm the role of animals identified in shrimp farms in relation to AHPND, such as crabs, mice (rats), and fish-eating birds. This has led to the hypothesis that animals in shrimp farms, general management, stock/postlarvae, and the other factors at the farm level are associated with AHPND in Bac Lieu Province. Overall, AHPND is a severe disease in shrimps that are caused by *Vibrio parahaemolyticus* with special plasmid-encoded with a toxin genome [2] and the insect-related binary toxin PirAB [14-16] (VP_AHPND_). This disease causes great economic losses annually and adversely affects not only the environment in shrimp farming areas in Bac Lieu but also in other provinces in Vietnam. Effective prevention of AHPND is very important for sustainable shrimp farming in Vietnam.

Therefore, research to identify the risk factors of AHPND is necessary and urgent. The objective of this study was to determine potential risk factors of AHPND at the farm level in Bac Lieu province, Vietnam.

## Materials and Methods

### Ethical approval andInformed consent

No ethical approval was required for this study. The shrimp farmers were voluntarily asked to participate in this study. The available information and interviews of shrimp farms were collected by face-to-face interview with the agreement of farmers and local authorities.

### Study areas and period

**Figure-1:**
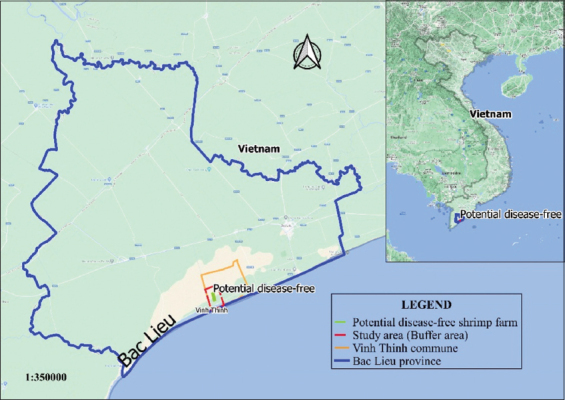
Buffer area in Vinh Thinh commune, Hoa Binh district, Bac Lieu Province, Vietnam [Map prepared in QGIS software].

The study was conducted in Vinh Lac and Vinh Tien villages of Vinh Thinh commune from March 2017 to December 2018. Vinh Thinh is one of the communes in Hoa Binh district, Bac Lieu Province, Vietnam, occupying 11,908.26 ha (Figure-1); most of the area is used for aquatic animal farming. Furthermore, this commune is one of the largest shrimp farming areas in Hoa Binh district. However, more than 200 ha/year is lost due to AHPND.

### Active surveillance program in buffer area

The repeated cross-sectional study was designed and commenced inMarch 2017 as a collaborative project between the Department of Animal Health (DAH) of Vietnam and Sub-Department of Livestock Production and Animal Health of Bac Lieu Province (Sub-DAH Bac Lieu) with field inspections carried out once per month. All the shrimp farms in the buffer area were listed and detailed information was obtained from a program undertaken in November 2016 and coded. For eachsampling round (1 round per month, a total 10 rounds in 2017), 30 shrimp farms were selected randomly from the list of shrimp farms. Thus, during monitoring, the selected farms in each round could be different.

Sampling in each round involved one pond if the shrimp farm had only a single pond or two ponds if the farm had two or more ponds using random sampling where necessary. Where possible, selected ponds were different in each round. Shrimps, water and sediment (environmental sample), and other crustaceans (if present) were sampled to test for diseases. For each farm, one pooled sample of shrimps and one pooled sample of the environment (water and topsoil) were taken from five different locations in each pond; there was one pool sample of crustaceans. The information collected on the samples and by the questionnaire was designed by the DAH.

The Laboratory of Regional Animal Health Office number VII under the DAH, accredited ISO 17025, conducted the following tests: *white spot syndrome virus* (WSSV, shrimp, and crustacean sample); AHPND (pooled shrimp and environment sample); *Yellow head virus-genome type 1* (YHV-type 1 and pooled shrimp sample); *Necrotizing hepatopancreatitis–bacteria* (NHP-B and pooled shrimp sample), and *Taura syndrome virus* (TSV and pooled shrimp sample) using real-time polymerase chain reaction (PCR) and a PCR protocol approved by the DAH. The real-time PCR method used to test AHPND was issued by the DAH based on the guidelines of the Manual of Diagnostic Tests for Aquatic Animals of World Organization for Animal Health (OIE).

As a result, 90 from a total of 183 shrimp farms in the study area were selected and 489 samples were tested for AHPND throughout the ten sampling rounds from March to December. Consequently, 93 specimens presented positive.

### Study population and study design

All shrimp farms in the buffer area were considered target populations to minimize any bias. Shrimp farms were investigated, listed, and followed up from 2016, with many shrimp farms being tested not only for AHPND but also for other diseases (WSSV, NHP-B, TSV, and HYV-type1) in 2017 and in subsequent years. Three shrimp farm models (intensive, semi-intensive, and extensive) were considered based on the density of shrimps, condition of the farm, and the food fed to the shrimps.

The matched case-control study at the farm level was designed and conducted in 2018 based on the results of the active surveillance program in the buffer area. Case and control farms were detected as below:

(1) Cases shrimp farms met two requests: (a) Had at least one pond that was positive for AHPND based on real-time PCR and (b) had tiger shrimps or white leg shrimps in at least one pond, showing at least one clinical sign of the hepatopancreas (HP) being pale to white, atrophied (shrinkage) and hard to crush, swollen and easier-to-break with a soft shell or with gut discontinuity or without contents and weak or sudden death of shrimps or both.

(2) Confirmed control farms were negative for AHPND based on real-time PCR and had no clinical signs during 2017. Where there was inadequate information to confirm a control farm, it was selected if AHPND had not occurred on the farm for 1 year. The controls had to meet all the conditions for matching, including the same farm model and species and similar farm size.

### Sample size

The number of samples for this study was calculated using the Epi-tool software (Table-1).

**Table 1:** Calculation of number of farms for case-control study using Epi-tool.

Parameter	Value
Expected proportion in controls	0.3
Assumed odds ratio	5.6
Confidence level	0.95
Power	0.8
Study type	Case-control study
Sample size per group	20
Total sample size (both groups):	40

### Data collection

Data were collected from an active surveillance program conducted in the buffer area of Vinh Thinh commune, Hoa Binh district, Bac Lieu Province in December2017 and from the face-to-face interview with farmers based on a questionnaire. The questionnaire was pre-tested before applying in the field to ensure adequate information was included and that it was practical.

The information collected consisted of four areas: (1) Common information: Name, address, phone number, farm code, and location; (2) construction and scale of farm: Water system, pond system (settling and cultivation ponds); (3) management and administration: farm model, pond and water management; origin and quality of stock; disinfection of water; and tools; chemical and drug use, animals present on the farm before any AHPND outbreak (wild animals and grazing animals); and (4) status of disease: onset, age of shrimps, species, mortality, clinical signs, the number of infected ponds, infected area, and culture information.

The two interviewers were trained, well known in the study area and had good relationship with farmers. They had also participated in designing and conducting the active surveillance program of the authority in 2017.

### Statistical analysis

Independent variables were separated into five groups: (1) Structure of farm; (2) prepared pond activities; (3) taking care and management of shrimp farm; (4) stock; and (5) grazing and wild animals on the farm. The quantitative variable was transformed to qualitative variable using the median. The outcome variable was the presence or absence of AHPND at the farm level.

Analysis of the risk factors was performed using two steps. In the first, bivariate analysis using condition logistic regression was applied to identify the variables having p≤0.2 at α=0.05; these were called potential factors; second, multivariable analysis with potential factors was carried out. Conditional logistic regression was used to evaluate the association of various risk factors with the risk of AHPND, presented as odds ratios (OR) with 95% confidence intervals. A factor having OR>1 and p≤0.05 were defined as a risk factor, while a factor having OR<1 and p≤0.05 were defined as a protective factor. Software used in the analysis was: Microsoft Office 365 (Microsoft Corporation, USA), Epi-tool (Ausvet, Australia), R program (R Core Team 2017; v.3.4.3 with survival package, R-Studio v.1.2.1335, Vienna, Austria) and Quantum GIS (QGIS Development Team 2017, v.2.18, Free Software Foundation. USA).

## Results

### Identifying case and control farms

**Figure-2:**
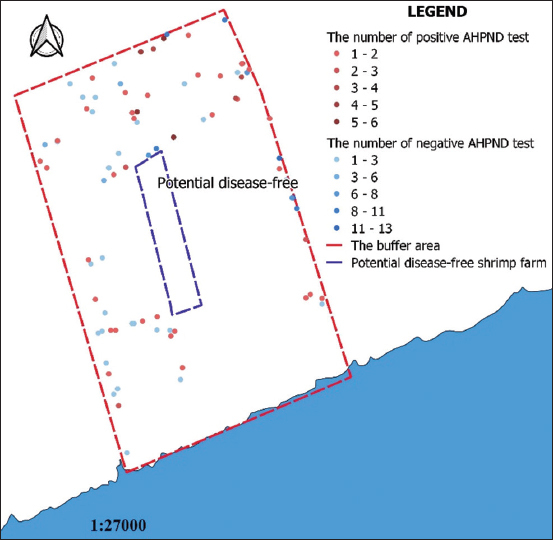
Location and the number of acute hepatopancreatic necrosis disease test of negative and positive shrimp farms [Map prepared in QGIS software].

In 2017, 90 farms were sampled to test for AHPND, WSSV, YHV-type, NHP-B, and TSV. As a result, the prevalence of *V. parahaemolyticus* was 19.02% at the sample level and 52.22% at the farm level (Table-2). In total, 47 shrimp farms were positive for AHPND, including 29 farms in Vinh Lac village and 18 farms in Vinh Tien village. In total, 43 shrimp farms were negative for AHPND in Vinh Lac (30) and Vinh Tien (13) villages during all of 2017. The locations of the shrimp farms are shown in Figure-2.

**Table 2:** Distribution of acute hepatopancreatic necrosis disease in buffer area.

Level	Village	Positive	Negative	Total	Prevalence (%)
Farm	Vinh Lac	29	30	59	49.15
	Vinh Tien	18	13	31	58.06
	Total	47	43	90	52.22
Sample	Vinh Lac	58	187	245	23.67
	Vinh Tien	35	209	244	14.34
	Total	93	396	489	19.02

AHPND=Acute hepatopancreatic necrosis disease

**Figure-3:**
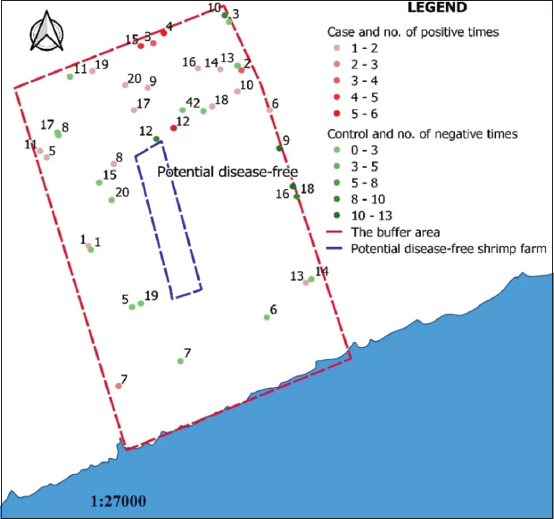
Location of matched case and control shrimp farms and the number of positive and negative with acute hepatopancreatic necrosis disease test [Map prepared in QGIS software].

In 2018, 134 farmers were interviewed, consisting of 71 farmers who had participated in the active surveillance program in 2017 and 63 new farmers (without AHPND testing) in the buffer area. From collecting and analyzing the database on AHPND testing and related information in 2017, together with the results of the investigation in 2018 and the definitions of case and control, 20 cases and 20 controls were identified and matched (Table-3 and Figure-3).

**Table 3:** Process of finding case and control shrimp farms.

Interview in 2018	Result of AHPND test in 2017	Result of interview in 2018 about AHPND	Conclusion of AHPND based on result of interview and AHPND test	Selected case and control for this study	Vinh Lac village (No. of shrimp farms)	Vinh Tien village (No. of shrimp farms)	Total
None	Negative	NA	NA	Removed	6	4	10
	Positive	NA	NA	Removed	6	3	9
	Subtotal				12	7	19
Yes	Negative	No disease	No disease	Confirmed control	6	6	12
				Removed	8		8
			Missing information	Removed	1		1
		Disease	Conflict	Removed	9	3	12
	Positive	No disease	Conflict	Removed	10	8	18
		Disease	Disease	Confirmed case	12	8	20
	None	No disease	No disease	Control	4	4	8
				Removed	23	12	35
		Disease	Disease	Removed	9	10	19
		NA	NA	Removed	1		1
	Subtotal				83	51	134
Total					95	58	153

AHPND=Acute hepatopancreatic necrosis disease

Distribution of AHPND in the 20 cases and the characteristics of the control farms are described in Table-4. There were 17, 2, and 1 cases from intensive, semi-intensive farm and extensive shrimp farms, respectively. Of these, 16 cases only related to tiger shrimp farms while four cases had both tiger and white-leg shrimps. Some shrimp farms had experienced AHPND at least twice in 2017. The sample totals of cases and controls tested for AHPND were 154 and 66, respectively.

**Table 4:** Characteristics of case and controls shrimp farms.

Parameter	Case	Control	Total	Percentage
Farm model				
Intensive	17	17	34	50
Semi-intensive	2	2	4	50
Extension	1	1	2	50
Total	20	20	40	50
Species infected by AHPND				
Tiger shrimp	16	16	32	50
White-leg shrimp	0	0	0	0
Both tiger and white-leg shrimp	4	4	8	50
Total	20	20	40	50
Farm size				
Area (ha)	52.5	44.2		
Mean	2.63	2.21		
SD	1.88	1.27		
Number of tested times	103	43	146	
Result of test (sample)				
Number of positive samples	45	0	45	100
Number of negative samples	109	66	175	62.29
Total sample	154	66	220	70

AHPND=Acute hepatopancreatic necrosis disease

The prevalence of AHPND at the pond level of the infected farms was 45.64%, the percentage of the infected area was 49.27% (Table-5). The cases group was not only tested but also had to show at least one clinical sign as presented in Table-6. The infected farms the following levels of clinical signs: Shrimps died suddenly (100%), discontinuous or empty gut (84.21%), atrophied HP (63.16%), and pale HP (89.47%), while only 26.32% of cases showed swollen and easily broken HP. Only 15.79% of the farmers reported that the shell of shrimps was soft.

**Table 5:** Distribution of acute hepatopancreatic necrosis disease at pond level in case group.

Parameter	Disease	No disease	Total	Prevalence (%)
Number of culture ponds (pond)	68	81	149	45.64
Culture are (ha)	24.27	24.99	49.26	49.27

**Table 6:** Clinical signs of acute hepatopancreatic necrosis disease of shrimp in case group.

Clinical sign	Yes	No	Total	Percentage
Death rate (>50%)	17	3	20	85.00
Shrimps died suddenly	20	0	20	100.00
Soft shell	3	16	19	15.79
Gut with discontinuous or no contents	16	3	19	84.21
Hepatopancreas pale	17	2	19	89.47
Hepatopancreas hard to crush	10	9	19	52.63
Hepatopancreas atrophied	12	7	19	63.16
Hepatopancreas swollen	5	14	19	26.32
Hepatopancreas easy to break	5	14	19	26.32

### Bivariable analysis

Variables were defined based on five groups: (1) Structure of shrimp farm; (2) prepared pond activities; (3) shrimp pond care; (4) stock factor group; and (5) grazing and wild animals on the farm. The variables were analyzed using bivariate analysis (Table-7). The quantitative variables were converted to qualitative variables based on the median. Conditional logistic regression was applied to find factors that had p≤0.2 using theR software (R core team 2017) with the survival package.

**Table 7:** Result of bivariable analysis using conditional regression at farm level.

Variable	Case	Control	OR	Coefficient	p-value
Structure of shrimp farm group					
Number of ponds in shrimp farm (median=5)					
Few (≤5 ponds)	10	14	0.33	-1.1	0.178
Many (>5 ponds)	10	6			
Farm size (median=2.1 ha)					
Small (≤2.1 ha)	9	11	<0.01	−20.2	1
Large (>2.1 ha)	11	9			
Water supply and drainage system					
Use same canal	19	19	1	<0.01	1
Separate canal	1	1			
Settling pond					
Yes	9	4	3.5	1.25	0.12
No	11	16			
Prepared pond activities group					
Sun-dried pond (median=62.5 days)					
Long (>62.5 days)	13	7	7	1.95	0.07
Short (≤62.5 days)	7	13			
Culture-free pond duration (median=90 days)					
Short (≤90 days)	17	15	0.5	−0.69	0.42
Long (>90 days)	3	5			
Removed topsoil after each crop					
Yes	20	20	NA	NA	NA
No	0	0			
Water filtered before entering farm					
Yes	19	16	>1000	20.2	1
No	1	4			
Chemical pond treatment before culturing					
Yes	19	18	> 1000	19.2	1
No	1	2			
Chemical disinfection of water before stocking					
Yes	19	18	> 1,000	19.2	1
No	1	2			
Caring for shrimp pond group					
Disinfected water during cultivated period					
Yes	19	17	> 1,000	20.2	1
No	1	3			
Disinfected tools during cultivated period					
Yes	13	15	> 1,000	21.2	1
No	7	5			
Use same tools for many ponds					
Yes	1	5	21.2	21.2	0.999
No	19	15			
Change water during cultivation period					
Yes	4	7	0.4	−0.92	0.27
No	16	13			
Periodic water disinfection					
Yes	17	16	1.5	0.41	0.66
No	3	4			
Use antibiotic for shrimps					
Yes	10	5	2.667	0.98	0.15
No	10	15			
Stock factor group					
Tested AHPND					
Yes	15	13	1.33	0.29	0.71
No	5	5			
Quarantine for stock					
Yes	16	11	2.67	0.98	0.15
No	4	9			
Use stock from more than one company					
Yes	9	4	2.25	0.81	0.18
No	11	16			
Grazing and wild animals on farm group					
Fish-eating birds land on culture area					
Yes	18	11	8	2.08	0.05
No	2	9			
Rats (mice) in culture area					
Yes	7	9	0.6	−0.51	0.48
No	13	11			
Crabs in culture area					
Yes	16	15	1.5	0.41	0.66
No	4	5			
Grazing animals beside culture area					
Yes	14	13	0.67	−0.41	0.66
No	6	7			
Grazing animals inside culture area					
Yes	6	5	1.25	0.22	0.74
No	14	15			

AHPND=Acute hepatopancreatic necrosis disease

In the first group, the structure of the shrimp farm included the number of ponds on the shrimp farm, farm size, water supply, and drainage system and a settling pond factor. Of these, only the number of ponds on the shrimp farm and farms with a settling pond had p<0.2 (0.18 and 0.12, respectively). In both the case and control, most (38/40 farms) used the same channel to supply and drain water.

In the prepared pond activities group, the results of the conditional analysis indicated that the sun-dried factor (median=62.5 days) had p=0.07 while, the factors that did not have p≤0.2 were: using chemicals (chlorine, saponin, and derris elliptica) to treat the bottom of pond (for killing crustaceans, eggs of fish, and other aquatic animals in the pond), culture-free pond duration, removing topsoil after each crop, disinfecting water before stocking (chlorine, benzalkonium chloride [BKC], and iodine).

Only one out of six factors in taking care of the pond during the cultivation period group had a p<0.2, namely, using antibiotics (p=0.15), with the other factors with higher p-values being water disinfection, using chemicals such as chlorine, iodine, and BKC to the sterilize water in the cultivation pond, changing water in the pond during cultivation and utilization and management of tools.

For factors related to the post-larvae group, the present study focused on tested AHPND before stocking, quarantine, and number of species in farms. In the stock group, two potential risk factors were identified as the quarantine variable and using stock from at least two companies, having p-values of 0.15 and 0.18, respectively.

For the last group (grazing and wild animals on farms), only the bird variable was a potential risk factor (p=0.05). This indicated that the percentage of farms where grazing other animals on the farm (both inside and outside the cultivation areas) or having wild animals such as mice or rats was high. Overall, seven factors with p≤0.2 were recognized in this study and subjected to subsequent multivariable analysis.

### Multivariable analysis

Seven variables which had p≤0.2 consisted; the number of ponds on the shrimp farm, settling pond, number of sun-dried days, using antibiotics during cultivation period, quarantine for PL, using stock from more than one company, and the fish-eating birds landing on culture area, were analyzed using multivariable analysis. During the analysis, factors having p>0.5 were removed one by one, starting with the largest value. Consequently, only one risk factor was recognized, namely, fish-eating birds landing on the farm during cultivation period, with OR=8.00 with 95% confidence level (CI) (1.00-63.96) and p=0.05 (Table-8).

**Table 8:** Results of multivariable analysis.

	OR	Lower	Upper	Coef.	p-value (>|z|)
Fish-eating birds land on culture area	8	1.00	63.96	2.08	0.05
Likelihood ratio test: 6.20, p*=*0.01					
Wald test: 3.84, p*=*0.05					
Score (Lagrange) test: 5.44, p*=*0.02					

OR=Odds ratio

## Discussion

The results of the matched case-control study showed that only the wild fish-eating birds on the culture areas had a significant association with AHPND. The risk for a farm increased 8 times if the fish-eating birds landed on the culture area. *V. parahaemolyticus* has been isolated not only from fish inhabiting brackish water [17], marine molluscan shellfish, crustaceans such as oysters and clams, and seawater and sediments [18-20] but also from bird fecal samples [21]. Wild birds (storks, kingfishers, snipes, and other fish-eating birds) caught fish, shrimps, *Polychaeta*, and other aquatic animals not only for immediate consumption but also could carry them back to their nests. Furthermore, these birds foraged on the large AHPD-infected area to capture AHPND-infected shrimps or any aquatic animals and could carry the VP_AHPND_ found in the water, soil, or aquatic animals to healthy shrimp ponds and other places, contributing to the transmission of *V. parahaemolyticus* and spreading AHPND in the culture area. This provided strong evidence to prove the recommendation of FAO in 2015 [22] regarding the role of predatory birds with AHPND. In addition, other studies proved that birds, such as seabirds and kingfishers, can carry causative agents such as infectious hypodermal and hematopoietic necrosis virus, TSV, and WSSV [23-26].

In addition, other animals such as crabs, mice, dogs, and chickens raised near or beside the cultivating area did not have a relationship with AHPND. The reason could have been that many farmers used a grid to surround their shrimp ponds and this restricted the intrusion of raised animals into their shrimp ponds. However, these domesticated animals could bypass the fences and harm the shrimp pond if the fences were not high enough. In addition, poor management might allow various animals, such as dogs, chickens, and swans, to graze inside the farm.

Based on the cross-sectional data from the MRD of Vietnam in 2012 under the TCP/VIE/3304 project of FAO, the case-control study showed that the risk factors at the farm level included a larger culture area, using the sun-dried method for cleaning the pond bottom during the pond preparation process and location near other farms and sharing the same water source were affected by AHPND [12]. However, our study did not produce the same results because of the different study designs and factors measured. In the current study, the case and control farms were matched according to models of shrimp farm, species, village, and farm size to explore the possible associations between disease and any variables for which the cases and controls were matched.

The current study did not prove the statistical significance of farms having a settling pond, drying the pond under sunlight for more than 62 days and using antibiotics during the cultivation period were risk factors of AHPND at the farm level. However, these might become risk factors if the sample size were large enough. The previous research in the MRD demonstrated that the sun-dried method for cleaning the pond bottom during the pond preparation process was a risk factor [12].

In the buffer area, some farmers used the settling pond as an extensive pond, or as a part of their lands to develop extensive ponds with the same water channel that supplied the intensive or semi-intensive ponds. It was possible that these channels were stocked with infected shrimps, but not knowing this, the farmers used the contaminated water to supply culture ponds, leading to an AHPND outbreak. In addition, VP_AHPND_ could enter the settling ponds through carriers such as fish-eating birds. Consequently, bacteria would enter the shrimp pond and result in AHPND occurring when the conditions were suitable for their development. To remove this risk, farmers should use settling ponds in an appropriate manner and apply good management to exclude or halt the intrusion of pathogens.

A study indicated that both virulent and non-virulent *V. parahaemolyticus* strains were resistant to several antibiotics [27], such as ampicillin, amikacin, kanamycin, rifampin, streptomycin, trimethoprim, and multiple antibiotics [28-33]. Accordingly, farmers might use antibiotics to prevent AHPND by mixing the antibiotics with shrimp feed and providing the antibiotics through the shrimp ponds. The antibiotics, if already in use, would not increase the cost of preventing *V. parahaemolyticus*, as they also killed other bacteria in the pond. In this case, farmers using antibiotics could be a risk factor regarding AHPND at the farm or pond level.

Finally, wild birds landing on culture areas were significantly associated with AHPND at the farm level. The fish-eating birds were usually attracted to the shrimp cultures and so need to be discouraged or prevented from intruding on such human activities. Other potential factors should also be further studied such as the quality of postlarvae, the number of species on the farm, crustacean, mud, or topsoil treatment and the chemicals used on the shrimp farm.

## Conclusion

Overall, there was a single risk factor identified, namely, wild birds landing on the farm during culturing (OR=8.00 with 95% CI [1.00-63.96], p=0.05). The study also found that farms having a settling pond, drying the pond using sunlight for more than 62 days, using antibiotics during the cultivation period and using stock from more than one provider were risk factors regarding AHPND at the farm level. Of these factors, sun-drying could become a risk factor if the sample size was large enough.

## Authors’ Contributions

HTN, AY, TR, and VB designed the study, revised the manuscript and given feedback on the contents. HTN analyzed the database and wrote the article draft. HTN, TNV and TTN conducted this study on the field and contributed to data collection. All authors read and approved the final manuscript.
